# Editorial: Methodological issues in consciousness research, volume II

**DOI:** 10.3389/fpsyg.2025.1585426

**Published:** 2025-04-02

**Authors:** Luca Simione, Antonino Raffone, Morten Overgaard, Axel Cleermans

**Affiliations:** ^1^Dipartimento di Scienze Umanistiche e Sociali Internazionali, Università degli Studi Internazionali (UNINT), Rome, Italy; ^2^Istituto di Scienze e Tecnologie della Cognizione, Consiglio Nazionale delle Ricerche (CNR), Rome, Italy; ^3^Department of Psychology, Sapienza University of Rome, Rome, Italy; ^4^Center of Functionally Integrative Neuroscience (CFIN), Department of Clinical Medicine, Aarhus University, Aarhus, Denmark; ^5^Center for Research in Cognition and Neuroscience (CRCN), ULB Neuroscience Institute (UNI), Université Libre de Bruxelles, Brussels, Belgium

**Keywords:** awareness, consciousness, methodological challenge, metacognition, perceptual awareness

The study of consciousness spans a vast array of domains, including perceptual awareness, cognition and metacognition, executive control, selfhood, sleep and dreaming, emotional competence, and empathy. It concerns both healthy states (e.g., meditation, aging, spiritual experiences) and pathological conditions (e.g., epilepsy, neglect syndromes, locked-in syndrome, minimally conscious states, anesthesia). Despite decades of interdisciplinary research, the fundamental nature and mechanisms of consciousness remain elusive (Dehaene, 2017; Seth and Bayne, 2022). Several key theoretical distinctions continue to fuel debate. For instance, the differentiation between phenomenal and access consciousness (Block, 1995), the pre-reflective (minimal) and the reflective (narrative) self (Gallagher and Zahavi, 2008), or between graded and all-or-none processing (Overgaard and Sandberg, 2021) remain unsettled. Similarly, methodological controversies persist: how to best measure awareness, how to establish its absence, and how to isolate the neural correlates of consciousness (Mashour et al., 2020). Recent debates also highlight the limitations of current paradigms in distinguishing necessary from incidental neural correlates (Koch et al., 2016). This Research Topic gathered recent contributions that address these theoretical and methodological challenges from diverse perspectives.

A core challenge in consciousness research is developing reliable measures that capture different levels and manifestations of awareness. Jia et al. introduce the Awareness Atlas, a novel self-report scale aimed at assessing what they called the “manifestations of consciousness”, i.e., behavioral, cognitive, and affective effects of different levels of awareness. This approach emphasizes the practical implications of measuring consciousness beyond its theoretical construct, particularly in areas like meditation research and clinical interventions. Watanabe and Moriguchi contribute to the long-standing debate on graded vs. all-or-none consciousness. By applying the Perceptual Awareness Scale (PAS) to an online discrimination task in children and adults, they provide evidence supporting a gradual emergence of conscious content. Notably, their findings suggest that while age does not significantly alter the emergence of subjective awareness, the gap between subjective experience and objective discrimination narrows over time.

Hulme et al. investigated whether report modality influences psychophysical sensitivity, a crucial issue in consciousness research that has received limited attention. Their study examines different report modalities in a perceptual discrimination task and reanalyzes previous data (Overgaard and Sørensen, 2004) to determine whether changes in report format affect perception itself. While their findings remain inconclusive, we advocated (together with the authors) for the necessity of further research into the relationship between report modality and conscious perception. Understanding if and how report modality interacts with perception mechanisms can deepen our general understanding of the perceptual conscious experience, allowing for a deeper (re-)evaluation of the widely accepted paradigm of the sensorimotor arc in favor of an alternative models, such as the one supported by report-dependent perceptual phenomena, in which different types of report manifest perceptual consequences.

Understanding how consciousness fluctuates across different states—such as wakefulness, sleep, and anesthesia—provides crucial insights into its mechanisms. Cecconi et al. propose a novel fMRI protocol to investigate sensory gating during disconnected dreaming states under propofol anesthesia. By combining neuroimaging with serial awakenings, their study offers a promising approach to identifying neural markers of disconnected vs. connected consciousness, with potential applications in anesthesia and disorders of consciousness.

The intersection of consciousness and emotion remains a crucial but overlooked area of the human experience. To fill this gap, van Wyk et al. employ sentiment analysis techniques—a branch of Natural Language Processing—to study emotional fluctuations in dreams. This methodological innovation provides an objective way to track the interplay between cognitive and emotional elements in dreaming, moving beyond traditional self-report approaches. With this new approach, they demonstrated how the emotional tone of dream content exhibits peaks and troughs across different dream segments. Instead, exploring the intensity of emotions and how it could shape our experience, Gómez-Emilsson and Percy pointed out the importance to take into consideration more seriously in research the incredible range of highs and lows which characterized such emotional experience. They challenge conventional models of emotional experience with their Heavy-Tailed Valence (HTV) Hypothesis. Contrary to standard models that assume a constrained valence range, their research suggests that the most intense emotional experiences (both pleasurable and painful) are orders of magnitude more extreme than previously assumed. This perspective has broad implications for research on wellbeing, self-reported happiness, and affective neuroscience.

Theoretical models of consciousness often hinge on specific philosophical assumptions, which can shape empirical research in subtle but profound ways. Usher et al. critique the Unfolding Argument (UA) against causal structure theories, arguing that it imposes restrictive constraints that may hinder scientific progress. They advocate for a phenomenology-centered approach to consciousness studies, emphasizing the primacy of subjective experience in grounding empirical investigations. Put phenomenology at the center of the scientific exploration of consciousness is not only important, but necessary for the authors, and we agree with them in this consideration on the limits of methods in consciousness studies which fail in linking human experience with neurophysiological and behavioral data. Consistent with this claim, Forti (a, b) contributes to the ongoing theoretical discourse on consciousness with two insightful papers that examine the structure of conscious experience from a phenomenological perspective. In the first paper [Forti (a)], he argues that the hierarchy of spatial belongings underlies the cohesive perception of early vision, proposing that conscious experience is organized in a way that mirrors brain structures. This hierarchical framework challenges reductionist accounts of perception and suggests that consciousness is best understood through the intrinsic relationships within experience itself. Similarly, in his second paper, Forti (b) critiques the traditional focus on qualia and the subjective “what it is like” focus of consciousness studies. He advocates shifting the explanatory target toward the structural and relational properties of phenomenal experience, particularly in early visual processing. By doing so, he provides a fresh perspective on the long-standing debate between higher-order and first-order theories of consciousness.

## Conclusion

These contributions align with the broader methodological challenges discussed in this editorial, particularly the difficulties in operationalizing and measuring consciousness beyond subjective reports. The studies collected in this Research Topic highlight the need for refining conceptual models that account for the intrinsic organization of conscious perception, reinforcing the idea that empirical research on awareness must be complemented by rigorous phenomenological analyses. Moreover, they reflect the diverse and interdisciplinary nature of contemporary consciousness research. From novel measurement tools and state-dependent investigations to emotion-consciousness interactions and theoretical refinements, these contributions advance our understanding of one of the most complex scientific challenges. While fundamental questions remain unresolved, these works illustrate the ongoing evolution of methodologies and theoretical perspectives necessary to tackle the enigma of consciousness. Future research will benefit from continued interdisciplinary dialogue, integrating insights from neuroscience, psychology, philosophy, and computational modeling to refine our grasp of awareness and its mechanisms.
